# Assessing the usability by clinicians of VISION: A hierarchical display of patient-collected physiological information to clinicians

**DOI:** 10.1186/s12911-017-0435-3

**Published:** 2017-04-14

**Authors:** Cubby L. Gardner, Fang Liu, Paul Fontelo, Michael C. Flanagan, Albert Hoang, Harry B. Burke

**Affiliations:** 1 59th Medical Wing, Science and Technology, JBSA-Lackland, TX USA; 2 National Library of Medicine, Bethesda, MD 20814 USA; 3 Cardiology Clinic, Walter Reed National Military Medical Center, Bethesda, MD USA; 4 F. Edward Hébert School of Medicine, Uniformed Services University of the Health Sciences, Bethesda, MD USA

**Keywords:** Visual display, Informatics, Patient-generated data, Home monitoring, Heart failure

## Abstract

**Background:**

The inability of patients to accurately and completely recount their clinical status between clinic visits reduces the clinician’s ability to properly manage their patients. One way to improve this situation is to collect objective patient information while the patients are at home and display the collected multi-day clinical information in parallel on a single screen, highlighting threshold violations for each channel, and allowing the viewer to drill down to any analog signal on the same screen, while maintaining the overall physiological context of the patient. All this would be accomplished in a way that was easy for the clinician to view and use.

**Methods:**

Patients used five mobile devices to collect six heart failure-related clinical variables: body weight, systolic and diastolic blood pressure, pulse rate, blood oxygen saturation, physical activity, and subjective input. Fourteen clinicians practicing in a heart failure clinic rated the display using the System Usability Scale that, for acceptability, had an expected mean of 68 (SD, 12.5). In addition, we calculated the Intraclass Correlation Coefficient of the clinician responses using a two-way, mixed effects model, ICC (3,1).

**Results:**

We developed a single-screen temporal hierarchical display (VISION) that summarizes the patient’s home monitoring activities between clinic visits. The overall System Usability Scale score was 92 (95% CI, 87-97), *p* < 0.0001; the ICC was 0.89 (CI, 0.79-0.97), *p* < 0.0001.

**Conclusion:**

Clinicians consistently found VISION to be highly usable. To our knowledge, this is the first single-screen, parallel variable, temporal hierarchical display of both continuous and discrete information acquired by patients at home between clinic visits that presents clinically significant information at the point of care in a manner that is usable by clinicians.

## Background

Accurate clinical information is essential for the optimal management of patients. Historically, information regarding the outpatient’s clinical condition at home was obtained during episodic clinic visits. During these visits patients provided subjective verbal recollections, sometimes supplemented by subjective paper-based logs and diaries [1]. However, these patient reports have been shown to be incomplete and inaccurate [2–5]. The inability of patients to accurately and completely recount their clinical status between clinic visits reduces the clinician’s ability to properly manage their patients [6].

One way to improve this situation is to collect objective patient information while the patients are at home. This approach has recently become feasible due to the introduction of mobile electronic devices that collect and transmit clinical information. These devices allow outpatients to collect their clinical information in an ecologically valid setting and provide objective information about their condition to their clinician during their clinic visit [7, 8]. A devise can collect one channel of information, e.g., just heart rate, or it can collect multi-channel information, e.g., both heart rate and respiratory rate. In either event, the information must be displayed. This research focuses on the display of multichannel objective information to the clinician.

There are several ways to display mobile multichannel information to the clinician [9]. One approach is to display all the continuous analog information sequentially or in parallel by channel, e.g., heart rate in one channel, respiratory rate in another channel. For example, Apple’s Health Dashboard shows separate parallel channels [10], Lifeline aligns the channels to a common time line [11], and Timeline aligns events in separate temporally aligned windows [12]. The main problem with this approach is that there is too much data to be viewed in real time.

Another approach is to summarize the information and present parameter estimates; however, the use of parameter estimates loses detailed information as the time intervals become larger. This makes the information manageable but it creates its own problems. First, it is not clear what the natural time intervals are. Second, the viewer cannot see small but clinically significant changes in the patient’s condition. Systems such as KNAVE-II [13], VISITORS [14], and Midgaard [15] dynamically scale the time interval and provide parameter estimate of summarized data to display quantitative data by summarizing detail as the user selects increasing temporal intervals (zooms out from seconds to minutes, hours, days, etc.). Finally, a limitation to dynamic display is that users need to dynamically zoom out and in to acquire the focus and context.

Ideally, the display would present the collected multi-day clinical information in parallel on a single screen, it would highlight threshold violations for each channel, and it would allow the viewer to drill down to any analog signal on the same screen, including the highlighted threshold violations, while maintaining the overall physiological context of the patient. All this would be accomplished in a way that was easy for the clinician to view and use.

We hypothesized that we could collect nocturnal data regarding five clinical variables, namely, body weight, systolic and diastolic blood pressure, heart rate, blood oxygen saturation, and physical activity, and that we could display this information in a temporal hierarchical manner, which we call VISION (**V**iew **I**ntegrated **S**creen **I**nformati**on**), that would be easy for the clinicians to learn and use. VISION is a display method that allows multi-channel analog information to be presented at a high level and which can display threshold violations – all on one screen in order to maintain the physiology context. The clinician can select any part of the display for more detailed examination, including those areas that have threshold violations. Furthermore, because the display is of parallel channels, the clinician can observe that there are threshold violations across channels at a specific point in time. In other words, we assessed the usability of a single screen, hierarchically organized, interactive temporal display of five-channel (physiological variable) information on a single screen.

We chose patients with heart failure because they experience many serious symptoms at home; symptoms that drive their readmission to the hospital. We chose nocturnal symptoms because heart failure patients are usually unable to recognize, respond to, and report nocturnal symptoms [16–20]. This paucity of information is a serious issue for the clinicians who management heart failure patients.

## Methods

This prospective study was conducted at the Walter Reed National Military Medical Center heart failure clinic in Bethesda, Maryland. The fourteen credentialed clinicians practicing in the heart failure clinic were invited to participate in this study and all agreed to do so. This represents the complete population of clinicians. They received iPads which they used to access VISION online. They were asked to review de-identified patients in the database and provide their impressions of usability by completing the validated System Usability Scale (SUS) instrument [21]. The variable of interest was the clinicians’ SUS score of the information display.

### Clinical information

**Fig. 1:**
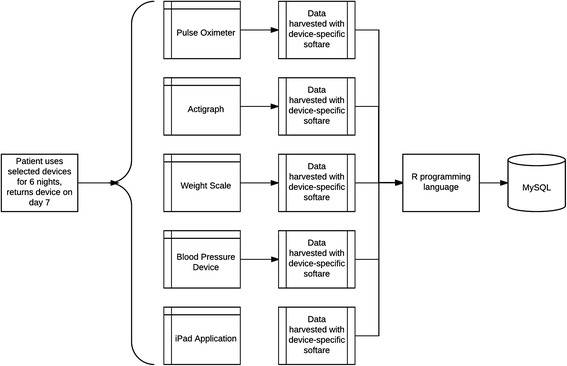
Data collection over six nights; device data is extracted with device-specific software and uploaded to the MySQL database using R programming language

We previously conducted a 39 heart failure patient study to assess patients’ ability to use mobile devices at home [22]. Experienced heart failure clinicians selected five clinical variables for their diagnostic and prognostic value in heart failure. These variables were consistent with the important variables in the heart failure literature. The clinical variables were: systolic and diastolic blood pressure, heart rate, blood oxygen saturation, physical activity, and body weight. Patients received five devices: blood pressure, pulse oximeter, actigraph, body weight scale, and an iPad. They used these devices at home for six consecutive nights. The procedure for patient data collection is shown in Fig. 1. We found that patients were able to use all of the devices and they rated the usability of all the devices higher than expected. Our study provided support for at-home patient-collected physiologic data. To our knowledge, this was the first study to assess the patient use and usability of mobile electronic devices by heart failure patients in their homes at night. These data were used as the input to VISION.

### Display

The goal of the display was to provide clinicians with a single screen, dynamic display that could be conveniently used at the point of care to quickly review the large amounts of information collected by patients between clinic visits and find clinical issues that required their attention. To optimize the fit of all information on to a single screen, time intervals were arranged hierarchically, showing 24-h overall data collection context, 8-h showing threshold violation segments, and 30-min showing second-level raw data.

**Fig. 2:**
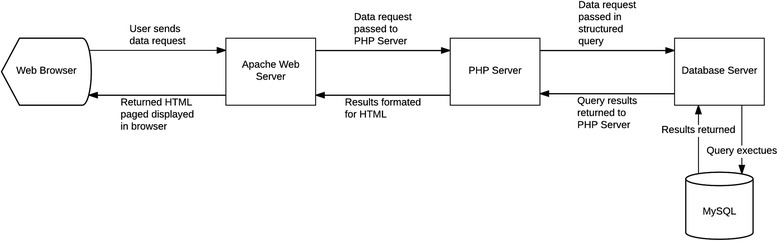
Process for clinician user retrieving specific patient information from the MySQL database via VISION web-based application

An open-source display was created using a web-based application employing a solution stack consisting of an Apache-based Web server, MySQL, and PHP. The dynamic readable display was created by scalable vector graphics (SVG), JavaScript, and AJAX. PHP server-side scripting language was combined with HTML to customize the interface [23]. Data from medical devices and subjective state assessment application (all linked to individual patients by an anonymous user ID) were uploaded to the MySQL database. The process for accessing patient data is illustrated in Fig. 2.

The large amount of data from medical devices required database design optimization. To improve speed and flexibility we constructed a data schema including two tables for each patient, one for episodic data and one for continuous data. Parsing the data into multiple tables allowed the database search to be significantly faster than from one single large table. To optimize loading, data to make the dynamic waveform layer was retrieved from the study server by AJAX technologies to build asynchronous Web apps. The process permitted updating portions of a Web page without reloading of the entire web page.

### Procedure

The heart failure clinicians logged into VISION and selected a patient from a list of all their patients. A 6-day record of an individual patient was displayed as 3 layers on one screen. Layer 1: 24-h day overview, sleeping period, feedback score, weight, and blood pressure measurement. The algorithm detected sleeping periods by checking the first and the last time slot that had either blood oxygen saturation (SaO2) or heart rate (HR) data measured during a 24-h period (usually 4 pm to 4 pm the following day). Layer 2: Sleeping period details, included SaO2, HR and activity were displayed in 3 rows. An algorithm to retrieve real activity was developed by adding a threshold on the average activity data every 30 min. Average activity data during a 30- min period above 12 was considered real activity. Layer 3 is a dynamic layer to show 30 min of data selected by the reviewing clinician from Layer 2. The waveforms for SaO2, HR and activity were presented as SVG format, for rendering graphical elements that can be infinitely scaled in size without losing resolution or clarity [24].

### Presentation

VISION was presented in the same way to all the clinicians. The clinicians opened VISION by clicking on its icon. They were instructed to go to the same patient and they were then instructed to view the same information for each patient. After viewing the identified patients, clinicians were allowed to manipulate the program at will. They were then asked to compete the System Usability Scale (SUS). All the clinicians were heart failure clinicians and all the patients were heart failure patients.

### System usability scale

The System Usability Scale (SUS) is a 5-point Likert rating scale that ranges from 1 = strongly disagree to 5 = strongly agree for each of ten items, the results are transformed to a 0 to 100 scale with an overall mean score of 68 (SD, 12.5) [21, 25, 26]. This transformation does not affect the truth-value of the scores. The SUS has been widely used for general usability testing because it has demonstrated reliability and internal consistency ranging from 0.85 to 0.90, and factor analysis shows that the factors load on two subscales, learnability and usability [26].

### Statistical methods

The clinicians’ System Usability Scale scores were tested against the expected score for “highly acceptable” of 68 (SD, 12.5) from the literature [21, 25, 27, 28] using student’s *t*-test. The subscale scores for usability and learnability were calculated according to the procedure described by Lewis and Sauro [26]. To assess consistency and absolute agreement we calculated the Intraclass Correlation Coefficient (ICC) using a two-way, mixed effect model, ICC (3,1), per the procedure described by Shrout and Fleiss (1979). Statistical tests were calculated using R, version 3.1.3 (Comprehensive R Archive Network, http://cran.r-project.org) and the significance value was set at < 0.05.

## Results

We assessed the creation and usability of a five-variable, temporal hierarchical display, VISION (**V**iew **I**ntegrated **S**creen **I**nformati**on**), which provides the clinician with a single-screen visual representation of the patient’s condition at home between clinic visits.

**Fig. 3:**
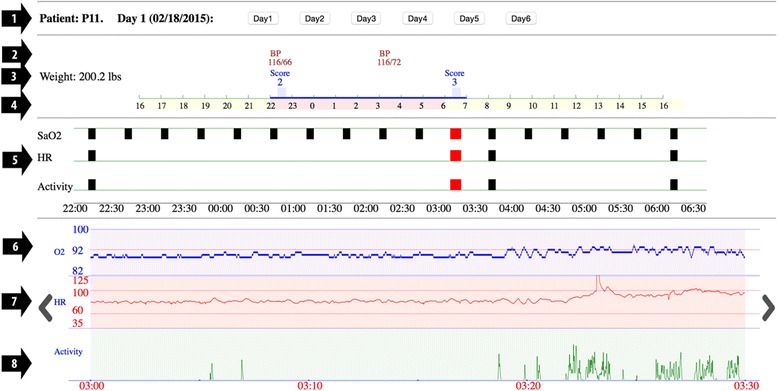
Electronic display of nocturnal heart failure information showing the interactive 24-h view (arrows 2–4), 8-h view (arrow 5), and 30-min view (arrow 6–8), arrayed onto a single screen

**Fig. 4:**
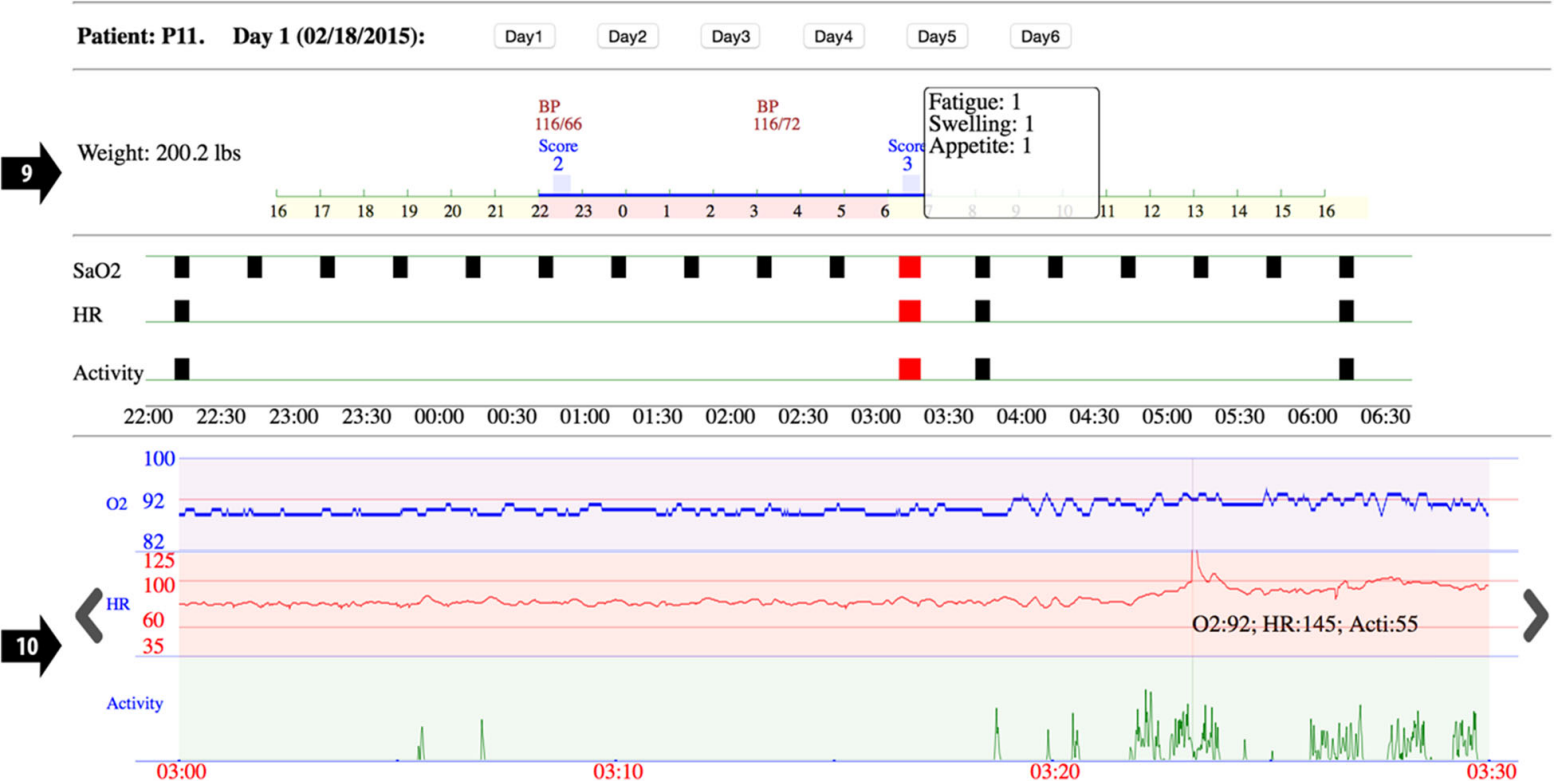
Additional detail showing subjective question set (arrow 9), and discrete values for 1-s level data on vertical cursor (arrow 10)

VISION was organized so that information at the top of the display had lower temporal granularity and information near the bottom of the display had higher temporal granularity. Temporal granularity refers to the selection of a temporally appropriate time scale [29]. The screen displayed all the variables using three stacked temporal dimensions: 24-h in hours, 8-h in minutes, and 30-min in seconds (Fig. 3). The dimensions were dynamically linked so that clicking on one dimension displayed the dimensions below it. The arrows are shown in Figs. 3 and 4 are for illustrative purposes only.

The top section of VISION has four lines. Arrow 1 points to the patient’s name and the day. Day 1 means the first day of the patient display and its associated date. Days 2–6 represent successive days for which there is data. We collected and displayed 6 days of information. In terms of the capability of the display, there can be any number of days; the line can scroll to the right.

Arrow 2 points to the blood pressure reading and the time it was taken. There are two blood pressure readings displayed on this screen. In terms of capability of the display, any number of blood pressure readings, and their associated times can be displayed.

Arrow 3 points to the weight and the subjective assessment scores, and times they were taken. Selecting the blue block associated with the subjective score provides additional detail for that score. In terms of capability of the display, any number of subjective scores, and their associated times can be displayed. These displayed subjective data are placeholders for patient collected subjective states.

Arrow 4 points to the 24-h time interval that comprises Day 1. The blue line represents the period of pulse oximeter wear. If the pulse oximeter were not worn, the blue line would be absent. The red band represents the 8-h time interval that is displayed in the section below. The line can be moved anywhere on the 24-h time interval and the section below will display that 8-h interval.

Arrow 5, the middle section, points to blocks, each of which represents a 30-min time interval. If the block is present it represents a violation of a threshold. There were thresholds for three variables, namely, oxygen saturation whose threshold is 92%, heart rate whose upper threshold is 100 beats per minute and lower threshold is 60 beats per minute, and physical activity whose upper threshold is 12 vector magnitude units. When one clicks on a block, the 30-min window for that block is shown in the section below and the blocks in the selected 30-min segment turn red.

Arrow 6 points to the continuous (per second) oxygen saturation. The display range is from 82 to 100%, with a threshold set at 92%. Putting a cursor over a point on the display show the exact reading. Arrow 7 points to the continuous (per second) heart rate. The display range is 35 to 125, with thresholds at 60 and 100. Arrow 8 points to the continuous (per second) physical activity. Arrows 6 – 8 are temporally aligned so that the clinician can see the correlation of oxygen saturation, heart rate, and physical activity.

Arrow 9 points to the subjective categories, and their values, that gave rise to the display subjective score (Fig. 4).

Arrow 10 points to a vertical cursor that provides exact values for the three variables blood oxygen saturation, heart rate and activity at each moment in time.

Fourteen clinicians participated in the evaluation of VISION; clinician characteristics are shown in Table 1. After using the display, clinicians completed the System Usability Scale (Table 2). The mean and 95% confidence interval for the System Usability Scale overall score was 92 (87, 97); this was significantly higher than expected, *p* < 0.0001. The means and 95% confidence intervals for usability and learnability subscale scores were 92 (86, 98) and 93 (87, 98), respectively, both of which were significantly higher than expected, *p* < 0.0001. We were also interested in the absolute agreement of clinicians’ System Usability Scale ratings. The Intraclass Correlation Coefficient of the clinician’s ratings was 0.89 (CI, 0.79, 0.97), which was significant, *p* < 0.0001.

**Table 1:** Demographics of clinician sample (*N* = 14)

Age (Mean, SD)	40.4 (8.6)
Gender n (%)	
Female	8 (57%)
Position n (%)	
Staff Physician	4 (29%)
Fellow	4 (29%)
Medical Resident	1 (7%)
Nurse Practitioner	4 (29%)
Physician Assistant	1 (7%)
Practice years n (%)	
Less than 1 year	0 (0%)
1–3 years	2 (14%)
4–6 years	2 (14%)
7–9 years	1 (7%)
10 or more years	9 (64%)

**Table 2:** Clinician system usability scale mean scores and Confidence Intervals (CI) for electronic display of clinical information

Score	Mean ± SD (95% CI)	*P*-value
Overall usability	92 ± 9 (87, 97)	*p* < 0.0001
Usability subscale	92 ± 10 (86, 98)	*p* < 0.0001
Learnability subscale	93 ± 10 (87, 98)	*p* < 0.0001

Clinicians commented that they found VISION very easy to use and informative. Several spontaneously remarked that the information would be useful during the visit. All clinicians were very quickly able to orient to the display and navigate the display independently.

## Discussion

We assessed the usability of a single screen, hierarchically organized, interactive temporal display of five-channel (physiological variable) information on a single screen. We found that the clinicians consistently gave VISION a high usability score. We expect that VISION will be used in the following way. Patients will be given the mobile devices, they will use them for six nights, and they will return to clinic with the devices in hand. The information on the devices will be downloaded in real time into VISION. By the time the clinician is ready to see the patient the data will be ready for display. The clinician will access the patient’s electronic health record and the VISION display. The clinician will examine and discuss the information displayed in VISION with the patient during the visit.

A common method for displaying physiologic data is temporally summarize and display the continuous data (e.g., heart rate, oxygen saturation, and movement), and to treat the episodic data (e.g., weight, blood pressure, subjective state) for display purposes as if it were continuous, and to display this information in a linear manner on parallel time lines (linear display). Alignment of data to a common time scale, the approach taken by such systems as Apple’s Health Dashboard [10], LifeLine [11], and Timeline [12], has been empirically demonstrated to be an easily perceived method of data visualization [30]. VISION also applies this principle, simultaneously displaying three time scales, but arranged hierarchically without summarization. Second-level data are displayed in the lower 30-min section, while the 8-h levels shows segments which contain threshold violations.

Visualizing high frequency data requires scaling of the time interval and reporting of parameter estimate dependent upon the user’s time scale selection. KNAVE-II is an early system to employ a specific computer subroutine to provide a parameter estimate for a specified time scale and return the day for display for an individual or small group of patients [13]. Additionally, VISITORS extend the technique to query across larger groups of patients [14]. Both cases allow users to linearly view select data element at a pre-specified time interval. Although modified to provide a dynamic update of the display through use of an interactive timeline, Midgaard provides users the ability to view multiple data elements scaled together on a common dynamic timeline [15]. VISION takes a different approach to provide focus plus context by providing information aligned to a common scale at three time intervals simultaneously and without summarizing the data.

VISION differs from other methods that provide parameter estimates on various time scales and linear display by providing high-level information on a single screen that can be drilled down to more granular information. The advantage of this approach is that the clinician can initially scan a large amount of temporal data in a short period, find areas of clinical concern, and then focus on just those areas of interest. Furthermore, VISION presents clinical information in their temporal relationships so that the clinician can observe the relationship between, for example, oxygen saturation and heart rate at any moment in time.

VISION recognizes that the appropriate time scale is variable, depending on the clinical and temporal context. VISION shows an overview of all the abnormal 30-min intervals that occurred over a selected 8-h interval. The clinician can select any 30-min interval, including an abnormal 30-min interval, within those 8 h and see the relevant variables displayed in parallel. In other words, the clinician can see what is happening to the patient over 8 h and see the relationship between heart rate, oxygen saturation, and activity related to that abnormality – all in the context of the discrete and subjective variables since they are time denominated as well. The use of a focus plus context approach has been shown to improve retrieval times from 21 to 36% [31, 32].

Another advantage of the VISION display is that it requires very little clinician interaction with the screen to obtain the needed information. Clinicians do not need to manipulate the time scale manually; the clinical information is displayed a day at a time, similar to how they would be a reference during a clinic visit. VISION can also display multiple days on a single screen.

Improvements in the devices that patients can use at home between clinic visits allows collection of ecologically valid data to provide clinicians with information at the patient’s next clinic visit that would not otherwise be available. The devices selected for this study were chosen for their ability to store data on the devices and for their usability. Clinical data collected by patients at home is more ecologically valid because patients collect it as they perform their normal activities of daily life. VISION aggregates ecologically valid clinical data in a way that provides medically useful information to the clinician when the patient returns to clinic. The VISION use case is for patients to the come to the clinic, receive the devices, take them home and use them for 6 days and nights, and return to the clinic on the seventh day. The clinical data is automatically downloaded and the clinician assesses the information with the patient at that return visit. This process can reoccur whenever there is a change in the patient’s clinical status.

A strength of this investigation was the use of a standardized and validated usability instrument. Klaassen and colleagues [33] reported that nearly two-thirds of 127 usability studies employed questionnaires to assess usability owing to their ease of use; however, this injects significant variability into usability assessment, particularly when questionnaires are customized or modified. The SUS has been extensively used and validated; the instrument produces a score between 0 and 100, with the global average score of 68 (SD, 12.5), and has demonstrated validity in small samples [25–28]. Moreover, since VISION is a novel display, SUS scores from this investigation provide a benchmark for future usability testing.

A potential study limitation is that its patient population was drawn from consecutive heart failure patients seen at one heart failure clinic, but we believe that these patients are representative of heart failure patients [34–37]. Another potential limitation is the relatively small number of clinicians, but we believe that the clinicians were representative of heart failure clinicians. A limitation of this study is that it only assessed subjective clinician usability judgments. Our next study will assess the clinical utility of VISION in terms of whether the clinicians consider the information useful and whether it changes their medical management. A limitation of this study was that it was specific to heart failure and it is not known if this method will generalize to other diseases.

## Conclusion

In summary, we created a single screen, hierarchically organized, interactive temporal display of five-channel (physiological variable) information on a single screen. Clinicians reported that they found the display to be easy to use and that it provided important clinical information regarding patients’ medical status. They found the simultaneous hierarchical temporally abstracted display to be an efficient way for them to rapidly evaluate the physiological information collected by patients at home at night between visits. To our knowledge this is the first single-screen, multi-variable temporal hierarchical display of physiological information acquired by patients at home between clinic visits. It has the potential to significantly improve the management of heart failure patients.

## Abbreviations

HR: Heart rate
ICC: Intraclass correlation coefficient
SaO2: Blood oxygen saturation
SUS: System Usability Scale
VISION: View Integrated Screen Information
